# MicroRNA treatment modulates osteogenic differentiation potential of mesenchymal stem cells derived from human chorion and placenta

**DOI:** 10.1038/s41598-021-87298-5

**Published:** 2021-04-07

**Authors:** Kulisara Marupanthorn, Chairat Tantrawatpan, Pakpoom Kheolamai, Duangrat Tantikanlayaporn, Sirikul Manochantr

**Affiliations:** 1 Department of Agricultural Technology and Development, Faculty of Agricultural Technology, Chiangmai Rajabhat University, Chiangmai, 50330 Thailand; 2 Division of Cell Biology, Department of Preclinical Sciences, Faculty of Medicine, Thammasat University, Pathumthani, 12120 Thailand; 3 Center of Excellence in Stem Cell Research, Thammasat University, Pathumthani, 12120 Thailand

**Keywords:** Cell biology, Stem cells

## Abstract

Mesenchymal stem cells (MSCs) are important in regenerative medicine because of their potential for multi-differentiation. Bone marrow, chorion and placenta have all been suggested as potential sources for clinical application. However, the osteogenic differentiation potential of MSCs derived from chorion or placenta is not very efficient. Bone morphogenetic protein-2 (BMP-2) plays an important role in bone development. Its effect on osteogenic augmentation has been addressed in several studies. Recent studies have also shown a relationship between miRNAs and osteogenesis. We hypothesized that miRNAs targeted to Runt-related transcription factor 2 (Runx-2), a major transcription factor of osteogenesis, are responsible for regulating the differentiation of MSCs into osteoblasts. This study examines the effect of BMP-2 on the osteogenic differentiation of MSCs isolated from chorion and placenta in comparison to bone marrow-derived MSCs and investigates the role of miRNAs in the osteogenic differentiation of MSCs from these sources. MSCs were isolated from human bone marrow, chorion and placenta. The osteogenic differentiation potential after BMP-2 treatment was examined using ALP staining, ALP activity assay, and osteogenic gene expression. Candidate miRNAs were selected and their expression levels during osteoblastic differentiation were examined using real-time RT-PCR. The role of these miRNAs in osteogenesis was investigated by transfection with specific miRNA inhibitors. The level of osteogenic differentiation was monitored after anti-miRNA treatment. MSCs isolated from chorion and placenta exhibited self-renewal capacity and multi-lineage differentiation potential similar to MSCs isolated from bone marrow. BMP-2 treated MSCs showed higher ALP levels and osteogenic gene expression compared to untreated MSCs. All investigated miRNAs (miR-31, miR-106a and miR148) were consistently downregulated during the process of osteogenic differentiation. After treatment with miRNA inhibitors, ALP activity and osteogenic gene expression increased over the time of osteogenic differentiation. BMP-2 has a positive effect on osteogenic differentiation of chorion- and placenta-derived MSCs. The inhibition of specific miRNAs enhanced the osteogenic differentiation capacity of various MSCs in culture and this strategy might be used to promote bone regeneration. However, further in vivo experiments are required to assess the validity of this approach.

## Introduction

Chronic and inflammatory bone diseases, including osteoporosis, are major causes of disability in the elderly that result in huge costs for the healthcare system^1^. With increasing life expectancy, the burden of age-related degenerative bone diseases will increase. Novel approaches, including cell-based therapies, are rapidly advancing in the treatment of degenerative bone diseases^2^.

Mesenchymal stem cells (MSCs) are multipotent stem cells that were first described in bone marrow as bone-forming progenitors by Friedenstein et al*.* in 1976^3^. These cells demonstrate self-renewal capacity and multi-lineage differentiation potential^4^. Both pre-clinical and clinical studies have emphasized their therapeutic potential because of their regenerative and immunomodulatory functions. They release several immunomodulatory factors that allow them to escape immune rejection for a sufficient time to exert their therapeutic actions^5^. In addition, MSCs are common progenitors of osteoblasts. During aging or under pathological stimuli, MSCs lose their osteogenic differentiation potential, resulting in progressive bone loss leading to increased skeletal fragility^6^. As a paradigm for tissue regeneration, MSCs, mostly from bone marrow, have been used for the treatment of bone degenerative diseases^7,8^. However, the harvesting procedure is invasive and the number of MSCs varies among donors^9^. Nowadays, MSCs can be isolated from various sources including chorion and placenta^10^. Chorion and placenta are good sources of MSCs because they are discarded biological samples. MSCs can be isolated from these tissues through a non-invasive procedure and can be prepared in huge stocks for clinical applications. A previous study demonstrated that MSCs from chorion (CH-MSCs) and placenta (PL-MSCs) could differentiate into osteoblasts^11–13^. Nevertheless, the osteogenic differentiation potential was not very efficient compared to MSCs derived from bone marrow (BM-MSCs)^10,14^. The precise mechanisms and reasons for this difference between bone marrow- and chorion- or placenta-derived MSCs are unclear.

Bone morphogenetic protein-2 (BMP-2), a member of the transforming growth factor-beta (TGF-β) superfamily, has been shown to facilitate bone repair. BMP-2 is crucial for inducing bone differentiation and bone formation. BMP-2 has attained attention in the field of bone repair due to its osteoinductive capacity^14^. BMP-2 is one of the growth factors that has been extensively used to induce osteogenesis in previous research and clinical applications^15^. However, data on BMP-2 facilitated osteogenesis by CH-MSCs and PL-MSCs is still limited.

MicroRNAs (miRNA) are endogenous small RNAs that exert vital regulating functions on various physiological processes including apoptosis, proliferation, and differentiation^15,16^. They negatively regulate gene expression through sequence-specific binding to target sites within the 3′-UTRs of mRNAs^17^ and also exhibit stage- and tissue-specific expression patterns during development^18,19^.

Osteogenesis is a delicately regulated process requiring temporal and spatial gene expression patterns that are finely controlled by hundreds of miRNAs^16,20^. Several miRNAs have been shown to promote or inhibit bone formation process. The previous studies reported that the up-regulation of miR-322, miR-34a, and miR10a expression levels promotes osteogenic differentiation of human BM-MSCs by enhancing the expression levels of several osteogenic transcription factors including runt-related transcription factors-2 (Runx-2) osterix (Osx), and osteocalcin (Ocn)^21,22^. In contrast, the overexpression of miR-31 suppressed the osteogenic differentiation of human BM-MSCs by down-regulating the expression levels of Runx-2, Osx, and Ocn while the inhibition of miR-31 increased the expression levels of those osteogenic promoting genes^21^. Among several transcription factors involved in the bone formation process, Runx-2 has been shown to play critical roles during the osteogenic differentiation of several osteoprogenitor cells including BM-MSCs^23^. Moreover, many signaling pathways that regulate bone formation, such as BMP-2 and Notch also exert their effect by modulating Runx-2 expression^24^. Due to its central role during the osteogenic differentiation process, we believe that the low osteogenic differentiation potential of CH-MSCs and PL-MSCs might be caused by the limited expression of Runx-2 during their osteogenic differentiation. However, the molecular events, especially the miRNAs, that control Runx-2 expression during the osteogenic differentiation of CH-MSCs and PL-MSCs are still poorly characterized. Although there is a previous report showing that miR-31, miR-106a, and miR-148a inhibited the expression of Runx-2, CBFB, and BMPs, and the down-regulation of these three miRNAs associated with the osteogenic differentiation of BM-MSCs^25,26^, the roles of these miRNA during the osteogenic differentiation of MSCs from other sources, including CH-MSCs and PL-MSCs are largely unknown. The present study therefore wants to compare the osteogenic differentiation capacity of CH-MSCs and PL-MSCs to that of BM-MSCs and investigates the role of miR-31, miR-106a, and miR-148a during the osteogenic differentiation of these MSC sources. We believe that the data obtained could be used to improve the osteogenic differentiation capacity of CH-MSCs and PL-MSCs and increase their potential as the alternative sources of MSCs for bone regeneration, in addition to BM-MSCs which are in limited supply.

## Materials and methods

### Isolation and characterization of MSCs

This study was approved by the Human Ethics Committee of Thammasat University No. 1 (Faculty of Medicine). All the experiments were carried out in accordance with the Declaration of Helsinki, the Belmont Report, CIOMS guidelines and ICH-GCP. All research subjects participated in this study after giving written informed consent. The chorion and placenta (n = 5) were obtained from pregnant woman after normal delivery. The tissues from human chorion and placenta were cut into small pieces and incubated with 1.6 mg/ml collagenase XI (Sigma-Aldrich, USA) and 200 mg/ml deoxyribonuclease I (Sigma-Aldrich, USA) at 37 °C for 4 h. Subsequently, the cells were washed with phosphate-buffered saline (PBS) and cultured in complete DMEM medium [Dulbecco’s Modified Eagle’s Medium (DMEM; GibcoBRL, USA) supplemented with 10% fetal bovine serum (FBS; Invitrogen, USA), 2 mM l-glutamine (GibcoBRL, USA), 100 U/ml penicillin and 100 μg/ml streptomycin (GibcoBRL, USA)]. Bone marrow (n = 5) were aspirated from ileac crest of normal healthy volunteers. Mononuclear cells (MNCs) from human bone marrow were isolated using density gradient centrifugation and cultured in the complete DMEM medium at a density of 1 × 10^5^ cells/cm^2^ in 25-cm^2^ tissue culture flasks (Costa, corning, USA). The cells were then incubated at 37 °C in a humidified atmosphere of 95% air and 5% CO_2_. After 3 days, the medium was gently removed and replaced with fresh medium. Medium change was performed twice a week. The cell morphology was observed and micrographs were taken using an inverted microscope (Nikon TS100, Japan). At confluence, the cells were washed with PBS and subsequently detached by incubation with 0.25% trypsin–EDTA (GibcoBRL, USA). The cells were collected and replated at a density of 1 × 10^4^ cells/cm^2^ for further expansion. MSCs from passages 3–6 were selected for further analysis.

### Surface marker analysis using flow cytometry

The expression of specific surface markers of each batch of cultured MSCs was examined using flow cytometry. After trypsinization, 5 × 10^5^ MSCs in 50 μl of phosphate buffered saline (PBS) were incubated with 5 μl of antibodies including the PE conjugated anti-CD34 antibody (BioLegend, USA), FITC conjugated anti-CD45 antibody (BioLegend, USA), PE conjugated anti-CD73 antibody (BioLegend, USA), FITC conjugated anti-CD90 antibody (BioLegend, USA) and FITC conjugated anti-CD105 antibody (BD Bioscience, USA), for 30 min at 4 °C in the dark. After washing with PBS, the cells were fixed with 1% formaldehyde for 15 min. For each marker, at least 2 × 10^4^ labeled cells were acquired using flow cytometry (FACSCalibur, Becton Dickinson, USA). The data were analyzed by the CellQuest software version 3.3 licensed to Thammasat University (https://timothyspringer.org/files/tas/files/cellquest-softwarereference.pdf).

### Adipogenic and osteogenic differentiation assays

MSCs from bone marrow, chorion and placenta were harvested to measure adipogenic and osteogenic differentiation potentials. For adipogenic induction, 7.5 × 10^4^ MSCs were seeded into 35-mm^2^ dishes containing adipogenic induction medium [DMEM supplemented with 10% FBS, 0.5 mM isobutyl-methylxanthine (Sigma-Aldrich, USA), 25 mM glucose, 1 μM dexamethasone, 10 μM insulin (Sigma-Aldrich, USA), 100 μM indomethacin (Sigma-Aldrich, USA)]. The medium was replaced every 4 days. After culturing for 28 days, the cells were washed with PBS and fixed with 4% formaldehyde for 30 min at room temperature Subsequently, the cells were stained with 0.3% Oil Red O (Sigma-Aldrich, USA) in isopropanol for 20 min. The cells were then washed with distilled water and the micrographs were taken using an inverted microscope (Nikon TS100, Japan).

For osteogenic induction, 4.5 × 10^4^ MSCs were seeded in 35-mm^2^ dishes containing 2 ml of osteogenic induction medium [DMEM supplemented with 10% FBS, 0.1 μM dexamethasone, and 50 μM ascorbic acid (Sigma-Aldrich, USA)]. The medium was replaced every 3 days. On day 7 of induction, 10 μM β-glycerophosphate (Sigma-Aldrich, USA) was added. For identification of calcification, the cells were subjected to alizarin red S staining (Sigma-Aldrich, USA) at day 28. After removing the medium, cells were rinsed with PBS and fixed with 4% formaldehyde at 4 ºC for 10 min. The cells were then washed twice with distilled water and stained with 40 mM alizarin red S (pH 4.2) for 30 min at room temperature. The micrographs were taken using an inverted microscope (Nikon TS100, Japan). For control, MSCs were cultured in a complete DMEM medium and processed similar to the cells in adipogenic or osteogenic differentiation medium, respectively.

### Assessment of MSC growth

To evaluate the growth characteristic of MSCs derived from chorion and placenta in comparison with MSCs derived from bone marrow, culture-expanded MSCs (passages 3–5) were trypsinized using 0.25% trypsin–EDTA and re-plated in a 24-well cell culture plate (Costar, Corning, USA) containing 1 ml of a complete DMEM medium at a density of 500 cells/cm^2^. The cells were maintained at 37 °C in a humidified tissue culture incubator with 5% CO_2_. Subsequently, the cells from 3 wells on each plate were harvested every 2 days to determine cell number using a hemocytometer. The mean of the triplicate cell counts for each day was calculated and plotted against culture time to generate a growth curve.

To measure the growth kinetics of MSCs, cultured MSCs were trypsinized using 0.25% trypsin–EDTA (viable cells were counted at passage 1 using a trypan blue exclusion assay). For each subsequent passage (passages 2–8), 5 × 10^3^ cells/cm^2^ were seeded into 24-well cell culture plates (Costar Corning, USA). After 48 h, the number of cells after each passaging step was used to calculate the total number of yielded cells and the doubling time was calculated according to the formula:

$$\begin{aligned} {\text{Population}}\;{\text{doubling}}\;{\text{number }}\left( {{\text{PDN}}} \right) & = {\text{log }}\left( {{\text{N}}_{1} /{\text{N}}_{0} } \right) \, \times \, 3.31 \\ {\text{Population}}\;{\text{doubling}}\;{\text{time }}\left( {{\text{PDT}}} \right) \, & = {\text{ cell}}\;{\text{culture}}\;{\text{time/PDN}} \\ \end{aligned}$$

(N_1_ = cell number at the end of cultivation, N_0_ = cell number at culture initiation).

The population doubling time assays were performed in triplicate for each isolated cell population. The mean of the triplicate cell counts for each passage was calculated and plotted against culture time to generate a population doubling time curve.

### Osteogenic differentiation of BMP-2 treated MSCs

To examine the effect of BMP-2 on osteogenic differentiation potential of MSCs derived from chorion and placenta, 9.5 × 10^3^ cultured MSCs were seeded into 24-well plates (Corning, USA.) containing osteogenic induction medium both with and without 100 ng/ml of BMP-2 (R&D Systems, USA). The cells were then incubated at 37 °C in a humidified atmosphere of 95% air and 5% CO_2_ for 3, 7, 14, 21, and 28 days. At specific time points, the degree of osteogenic differentiation was measured by alkaline phosphatase staining using 5-Bromo-4-chloro-3-indolyl phosphate (BCIP)/nitro blue tetrazolium (NBT) (Sigma-Aldrich, USA). After washing with PBS, the cells were fixed with 4% formaldehyde at 4 °C for 5 min. Subsequently, BCIP/NBT liquid substrate was added into each well and incubated at room temperature for 30 min. After removing the staining solution, the cells were washed twice with distilled water. Micrographs were taken immediately on an inverted microscope (Nikon TS100, Japan). MSCs cultured in complete DMEM medium served as negative controls.

### Measurement of alkaline phosphatase activity

The quantitative alkaline phosphatase activity assay was performed using the SensoLyte pNPP Alkaline Phosphatase Assay Kit (AnaSpec, USA). Briefly, the treated MSCs of each condition on day 3, 7, 14, 21, and 28 were washed with 1 × assay buffer. Subsequently, 200 µl of 0.02% Triton X–100 in 1 × assay buffer was added and the adherent cells were scraped off. The cell lysate was incubated at 4 °C for 10 min with shaking. Then, the cell lysate was centrifuged at 2500×g for 10 min at 4 °C to collect the supernatant for alkaline phosphatase activity assay. After centrifugation, 50 µl of supernatant was added into each well of 96-well plates (Costar, Corning, USA). A serially diluted alkaline phosphatase solution, 0–10 ng/ml in 1 × assay buffer, was used as a standard. Then, 50 µl of p-nitrophenyl phosphate (pNPP) substrate solution was added into each well, mixed by gently shaking the plate for 30 s and incubated at room temperature for 60 min. Subsequently, ALP activity was measured on a microplate reader (BioTex, USA) using absorbance at 405 nm. The ALP activity in each sample was calculated by comparing the measured OD values against the standard curve. Total protein levels were determined by the Bradford assay (Bio-Rad, USA). ALP activity was calculated as the amount of phosphorylated nitrophenol release in ng/μmol/min and was further normalized to the total cellular protein. Each assay condition was assessed in triplicate. MSCs cultured in complete DMEM medium were used as a control.

### Quantitative real-time RT-PCR

To quantify the degree of osteogenic differentiation after BMP-2 treatment, real-time RT-PCR was performed using total RNA extracted from each culture condition on days 3, 7, 14, 21, and 28. The expression of the osteogenic marker genes, Runt-related transcription factor 2 (Runx-2), osterix (Osx) and osteocalcin (Ocn), were analyzed. For each sample, glyceraldehyde-3-phosphate dehydrogenase (GAPDH) was used as a housekeeping gene. Three independent samples were performed. Total RNA was isolated from each sample using the PureLink RNA Mini Kit (Invitrogen, USA) according to the manufacturer’s instruction. The RNA concentration was measured using a NanoDrop 2000 spectrophotometer (Thermo Scientific, USA). The messenger RNAs were reverse transcribed to cDNA using Superscript III First-Strand Synthesis Kit (Invitrogen, USA). The polymerase chain reaction amplification of the cDNA products was performed using the SYBR Green PCR Master Mix (Applied Biosystems, USA). The primers listed: Runx-2, forward: 5′-GACAGCCCCAACTTCCTGT-3′, reverse: 5′-CCGGAGCTCAGCAGAATAAT-3′ (159 bp); Osx, forward: 5′- TGCTTGAGGAGGAAGTTCAC -3′, reverse: 5′- CTGCTTTGCCCAGAGTTGTT -3′ (114 bp); Ocn, forward: 5′- CTCACACTCCTCGCCCTATT -3′, reverse: 5′- TCAGCCAACTCGTCACAGTC -3′ (245 bp). The amplification was performed on the StepOnePlus Real-Time PCR System (Applied Biosystems, USA) using 40 cycles of amplification (denaturation at 95 °C for 10 s, annealing at 60 °C for 10 s and extension at 72 °C for 20 s) after an initial activation at 95 °C for 10 min. Melting curves were assessed to ensure a single product was quantified. Each sample was examined in triplicate and the mean value was calculated. The mRNA expression level was analyzed by the comparative threshold cycle value (ΔΔCT) method using StepOne Software version 2.3 (https://www.thermofisher.com/id/en/home/technical-resources/software-downloads/StepOne-and-StepOnePlus-Real-Time-PCR-System.html) and presented as fold change relative to untreated controls.

### Expression of microRNAs involved in osteogenic differentiation

The expression of microRNAs involved in osteogenic differentiation was examined using quantitative real-time polymerase chain reaction (qRT-PCR). Briefly, BM-MSCs, CH-MSCs and PL-MSCs at passages 3–5 were cultured in 25cm^2^ tissue culture flasks (Costar Corning, USA) with complete medium and allowed to adhere to the plate overnight. Subsequently, the medium was removed and osteogenic differentiation medium both with and without 100 ng/ml of BMP-2 was added. MSCs cultured in complete DMEM medium were used as a control. Total RNA was extracted from the cultured MSCs using TRIzol reagent (Ambion, USA) at days 3, 7, 14, 21, and 28. MicroRNAs were reverse transcribed into cDNA using the TaqMan MicroRNA Reverse Transcription Kit (Applied Biosystems, USA). The reverse transcription was carried out in a MyCycler Thermal Cycler (Bio-Rad, USA). The reaction mixtures were incubated at 16 °C for 30 min, then at 42 °C for 30 min, and then inactivated at 85 °C for 5 min. The expression levels of hsa-miR-31-5p, hsa-miR-106a-5p, hsa-miR-148a-5p and U6 were analyzed by qRT-PCR. The mature miRNA sequences are shown in Table 1. The qRT-PCR samples were then prepared using TaqMan Universal PCR Master Mix II (Applied Biosystems, USA). The miRNA-specific primers were included in TaqMan MicroRNA Assays (Applied Biosystems, USA). The mixtures were first incubated at 95 °C for 10 min to activate the enzyme, and then they entered a cycle of qRT-PCR (denaturation at 95 °C for 15 s, and then annealing/extension at 60 °C for 60 s). The cycle was run 45 times. For quantitative calibration and normalization, the expression of U6 was used as an endogenous control. The baseline and threshold values were set and the comparative threshold cycle value (ΔΔCT) method was applied to quantify the miRNA expression level using StepOne Software version 2.3 (https://www.thermofisher.com/id/en/home/technical-resources/software-downloads/StepOne-and-StepOnePlus-Real-Time-PCR-System.html) and presented as the relative miRNA expression level.

**Table 1:** The mature miRNA sequence.

miRNA	Mature miRNA sequence
hsa-miR-31-5p	AGGCAAGAUGCUGGCAUAGCU
hsa-miR-106a-5p	AAAAGUGCUUACAGUGCAGGUAG
hsa-miR-148a-5p	AAAGUUCUGAGACACUCCGACU
U6	GTGCTCGCTTCGGCAGCACATATACTAAAATTGGAACGATACAGAGAAGATTAGCATGGCCCCTGCGCAAGGATGACACGCAAATTCGTGAAGCGTTCCATATTTT

### Transfections with miRNA inhibitors

To explore the effect of miRNAs on the osteogenic differentiation potential of MSCs derived from chorion and placenta, MSCs were seeded into a 6-well plate (Costar Corning, USA) containing complete DMEM medium and allowed to adhere to the plate overnight. Subsequently, the medium was removed and the osteogenic induction medium was added. For miRNA transfection, 10 nM of each miRNA inhibitor (miR-31 inhibitor, miR-106a inhibitor, and miR-148a inhibitor) diluted in osteogenic induction medium was mixed with Lipofectamine 3000 Transfection Reagent (Invitrogen, USA) and incubated for 10 min at room temperature. Then, the transfection complexes were added to the cultures. MSCs transfected with 10 nM FAM-labeled miRNA negative control #1 were used as a negative control. At days 3, 7, 14, and 21 of incubation, total RNA was extracted and the miRNAs expression levels were determined using quantitative real-time RT-PCR as mentioned above. To monitor the level of osteogenic differentiation after miRNA inhibitor transfection, ALP staining and ALP activity assay were performed on day 3, 7, 14, and 21. The micrographs were taken using an inverted microscope (Nikon TS100, Japan). Besides, the expression of osteogenic marker genes was also examined using qRT-PCR.

### Microscope imaging

The visualizations were performed in the Central Laboratory Research Unit, Faculty of Medicine, Thammasat University, Thailand.

### Statistical analysis

The data are presented as mean ± standard error of the mean (SEM). Statistical analysis was performed using the paired T-test for paired samples. A *P-value* of less than 0.05 was considered as statistically significant. The correlation between the osteogenic marker genes and miRNA expression was determined using Pearson's Correlation.

### Ethical approval and consent to participate

This study was approved by the Human Research Ethics Committee of Thammasat University No. I (Faculty of Medicine) and is in accordance with the declaration of Helsinki and Belmont report. All samples were obtained from donors with written informed consent.

## Results

### Characteristics of mesenchymal stem cells derived from chorion and placenta

**Figure 1:**
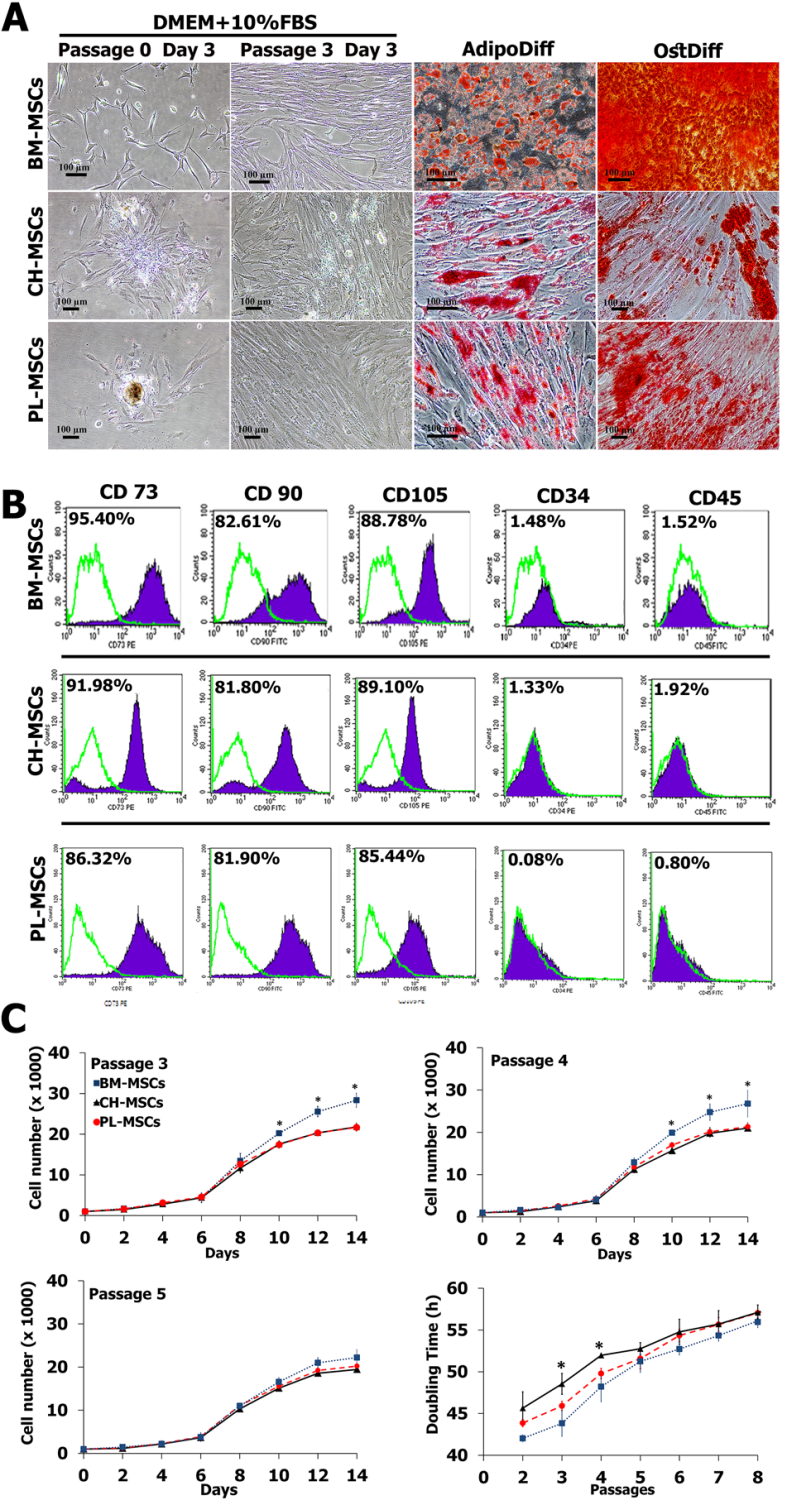
The characteristics of MSCs isolated from human chorion (CH-MSCs) and placenta (PL-MSCs) in comparison with MSCs isolated from human bone marrow (BM-MSCs). (**A**) The fibroblast-liked morphology and differentiation potential of CH-MSCs, PL-MSCs, and BM-MSCs. (**B**) Flow cytometry demonstrated the expression of typical MSC markers in CH-MSCs and PL-MSCs compared to BM-MSCs. (**C**) Growth characteristics of CH-MSCs and PL-MSCs compared to BM-MSCs. Data are presented as mean±standard error of the means (SEM). Scale bar = 100 μm.

MSCs were generated from human bone marrow, chorion and placenta. A day after initial plating, some cells adhered to the culture flask and exhibited fibroblast-like morphology. After removing non-adherent cells at day 3, the loosely adherent aggregated proliferating cells were observed under the inverted microscope (Fig. 1A). They displayed a spindle-shaped, fibroblast-like morphology. These MSCs were a continually cultured and homogeneous population of fibroblast-like cells, and 80% confluence was observed within 10 days after initial seeding. After passage 3, MSCs from bone marrow, chorion and placenta could rapidly proliferate and reached 80–90% confluence within approximately 3 days after seeding (Fig. 1A).

The differentiation potentials of BM-MSCs, CH-MSCs and PL-MSCs were investigated by induction in adipogenic and osteogenic induction media for 28 days, respectively (Fig. 1A). The spindle-shaped BM-MSCs, CH-MSCs and PL-MSCs cultured in adipogenic induction medium developed into bulky cells with many lipid droplets in their cytoplasm. These lipid droplets were positive for Oil Red O staining, which indicated the adipogenic differentiation efficiency of both CH-MSCs and PL-MSCs (Fig. 1A). Control MSCs cultured in complete DMEM medium showed no evidence of adipogenic differentiation and were negative for Oil Red O staining. To investigate the osteogenic differentiation potential of BM-MSCs, CH-MSCs and PL-MSCs, MSCs at passage 4 were cultured in an osteogenic induction medium. After 28 days of induction, the spindle-shaped BM-MSCs, CH-MSCs and PL-MSCs became flattened and broadened with increasing time of induction. They showed extracellular calcium deposits that were positive for alizarin red S staining (Fig. 1A). The MSCs from untreated control cultures showed no extracellular calcium deposits and were negative for alizarin red S staining. It should be noted that CH-MSCs and PL-MSCs need about 28 days for differentiation into an osteoblastic lineage.

Although CH-MSCs and PL-MSCs had lower osteogenic differentiation capacity compared with BM-MSCs, the immunophenotypes of these two MSC sources were similar to that of BM-MSCs. The expression levels of CD34, CD45, CD73, CD90, and CD105 of CH-MSCs and PL-MSCs, as determined by flow cytometry, were not significantly different from that of BM-MSCs (Fig. 1B). In addition, CH-MSCs and PL-MSCs also have the same proliferative capacity, as determined by the growth kinetic assay, as that of BM-MSCs (Fig. 1C). Taken together, we believe that the lower osteogenic differentiation capacity of CH-MSCs and PL-MSCs as compared with the BM-MSCs is unlikely to arise from the lower number of naïve MSC population in these two MSC sources.

### Growth characteristics of MSCs derived from chorion and placenta

The growth characteristics of MSCs derived from chorion and placenta in comparison with bone marrow were evaluated every 2 days up to 14 days in culture. The results showed that the proliferative capacity of CH-MSCs and PL-MSCs was similar to that of BM-MSCs during the first 6 days (Fig. 1C). Subsequently, the number of BM-MSCs, CH-MSCs and PL-MSCs showed a dramatic increase from after day 8 until day 14 in culture. During passages 3–4, BM-MSCs had a significantly higher proliferative capacity than CH-MSCs and PL-MSCs. Starting from 1 × 10^3^ cells at day 0, BM-MSCs could expand about 30-fold whereas CH-MSCs and PL-MSCs could expand about 20-fold within 14 days. Nevertheless, the proliferative capacity of BM-MSCs from passage 5 onward was not significantly different from those of CH-MSCs and PL-MSCs. (Fig. 1C).

The population doubling time of BM-MSCs appeared to be significantly lower than that of CH-MSCs and PL-MSCs during passages 2–4, indicating a higher proliferative rate of BM-MSCs than CH-MSCs and PL-MSCs during early passages. Nevertheless, the population doubling time in further passages showed a similar pattern (Fig. 1C). Based on these results, BM-MSCs, tended to double their populations at an average of 49.79 ± 5.32 h while CH-MSCs and PL-MSCs exhibited the doubling time of 52.37 ± 4.08 h and 51.17 ± 4.97 h, respectively.

### Expression of alkaline phosphatase after BMP-2 treatment

**Figure 2:**
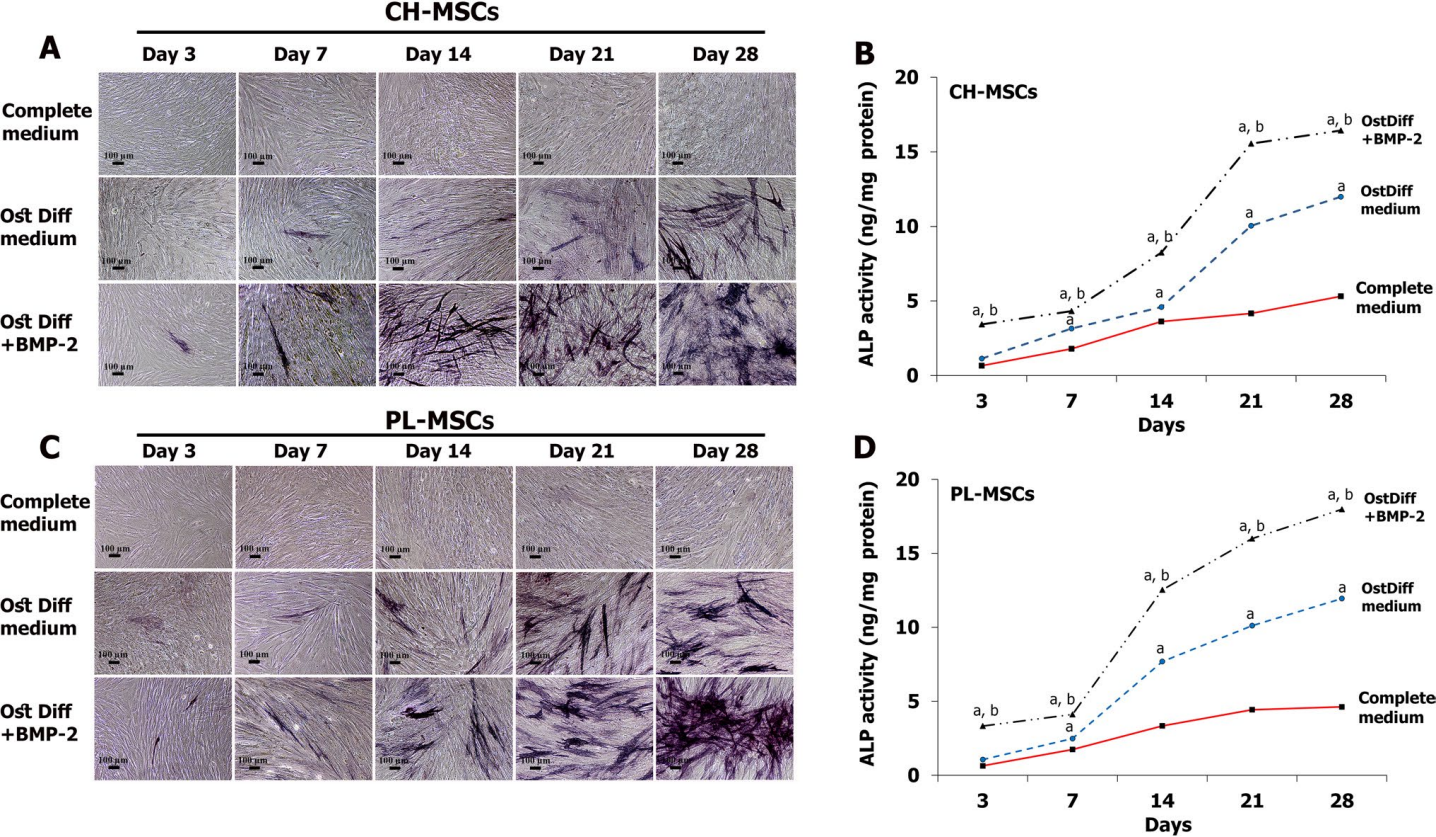
Alkaline phosphatase (ALP) cytochemical staining of CH-MSCs and PL-MSCs cultured in osteogenic differentiation medium with or without BMP-2. (**A**,**C**) ALP cytochemical staining at different time points shows an increase in ALP activity (deep blue) during osteogenesis. (**B**,**D**) ALP enzyme activity assay shows a configuration similar to the ALP staining assay. Data calculated from three independent experiments are presented as mean± SEM. *p*<0.05 compared to MSCs cultured in complete DMEM medium marked as “a”. *p*<0.05 compared with MSCs cultured in osteogenic differentiation medium (OstDiff medium) marked as “b”. Scale bar = 100 μm.

To study the effect of BMP-2 on the osteogenic differentiation potential of CH-MSCs and PL-MSCs, CH-MSCs and PL-MSCs were cultured in osteogenic induction medium both with and without BMP-2 for 28 days. The expression of ALP was evaluated by cytochemical staining on days 3, 7, 14, 21, and 28 after seeding. CH-MSCs and PL-MSCs cultured in complete DMEM medium served as a negative control. The results revealed that CH-MSCs and PL-MSCs that were treated with BMP-2 had a higher ALP expression than the untreated group (Fig. 2A, C). The expression of ALP in BMP-2 treated MSCs had progressively increased and reached a maximum level at day 28. In contrast, CH-MSCs and PL-MSCs cultured in the complete DMEM medium were negative for ALP staining throughout the entire culture period. Although the ALP expression levels of the untreated groups also steadily increased toward the end of culture, the levels were much lower than those of BMP-2 treated groups (Fig. 2A, C).

The quantitative ALP activity assay is consistent with the qualitative ALP staining. CH-MSCs and PL-MSCs treated with BMP-2 had higher ALP activity than the untreated group at every examined time point. The ALP activity of both CH-MSCs and PL-MSCs treated with BMP-2 had increased by day 28. ALP activity in PL-MSCs treated with BMP-2 increased notably from day 14 onwards, and ALP activity in CH-MSCs treated with BMP-2 showed a similar increase from day 21 onwards. (Fig. 2B, D).

### Expression of osteogenic genes after BMP-2 treatment

**Figure 3:**
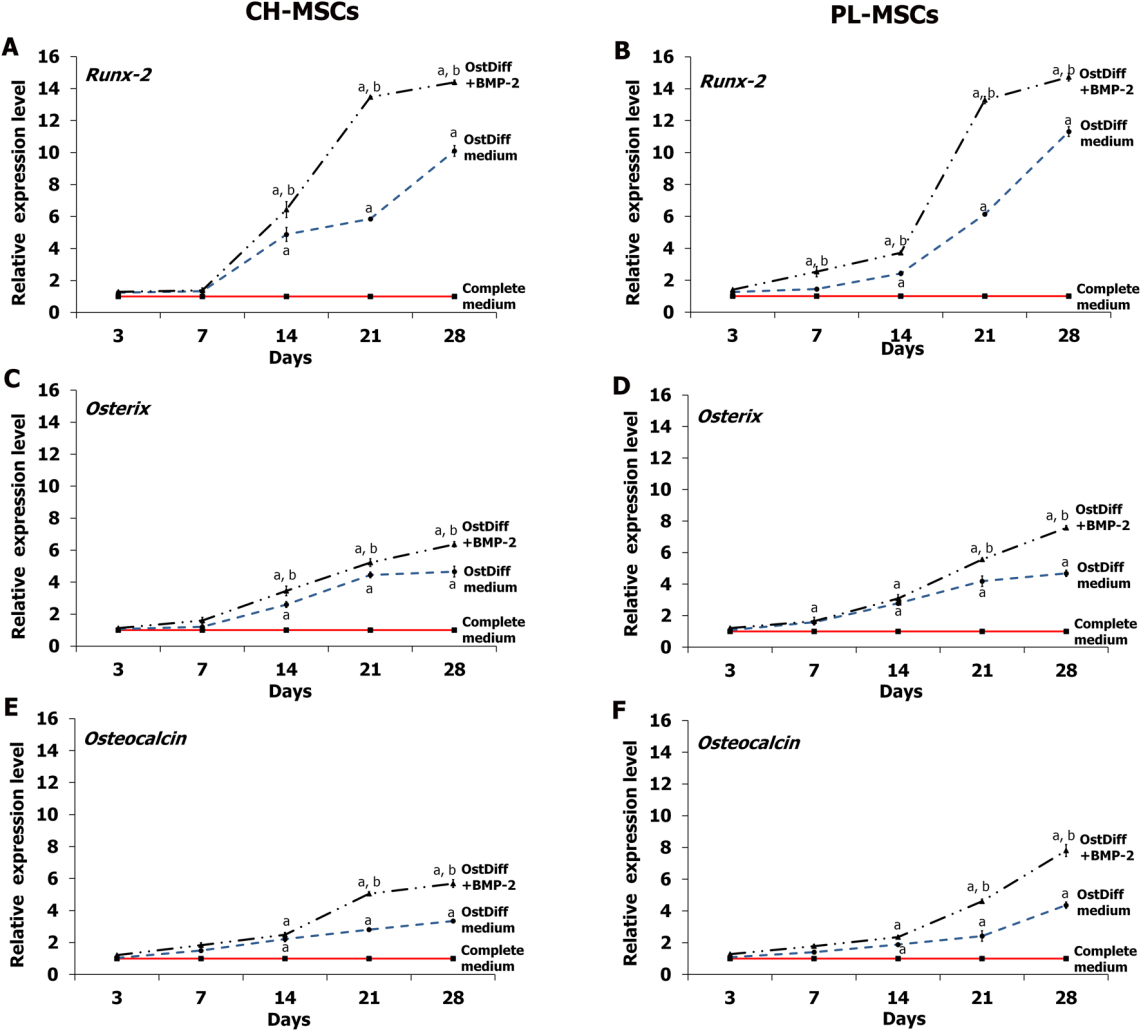
The expression of osteogetic markers in CH-MSCs (**A**,**C**,**E**) and PL-MSCs (**B**,**D**,**F**) cultured in osteogenic differentiation medium with or without BMP-2. Real-time RT-PCR analysis of Runx-2, Osx, and Ocn expression relative to GAPDH was performed at the indicated time points. *p*<0.05 compared to MSCs cultured in complete DMEM medium marked as “a”. *p*<0.05 compared to MSCs cultured in osteogenic differentiation medium (OstDiff medium) marked as “b”.

The expression of osteogenic genes, including Runx-2, Osx, and Ocn, in CH-MSCs and PL-MSCs, treated with BMP-2 compared to an untreated group was determined by quantitative real-time RT-PCR at the same time points as the ALP activity assay. The results revealed that the expression of Runx-2 in CH-MSCs (Fig. 3A) and PL-MSCs (Fig. 3B) in both the treated and the untreated groups steadily increased and reached their highest level on day 28, but the BMP-2 treated MSCs showed higher expression levels than the untreated MSCs. Similar to Runx-2, the expression levels of both Osx and Ocn in CH-MSCs and PL-MSCs in both the treated and untreated groups gradually increased throughout the entire culture period and reached their highest points at the end of culture period (Fig. 3C–F), but the expression levels were higher in the BMP-treated groups.

### The expression of miR-31, miR-106a, and miR-148a after BMP-2 treatment

**Figure 4:**
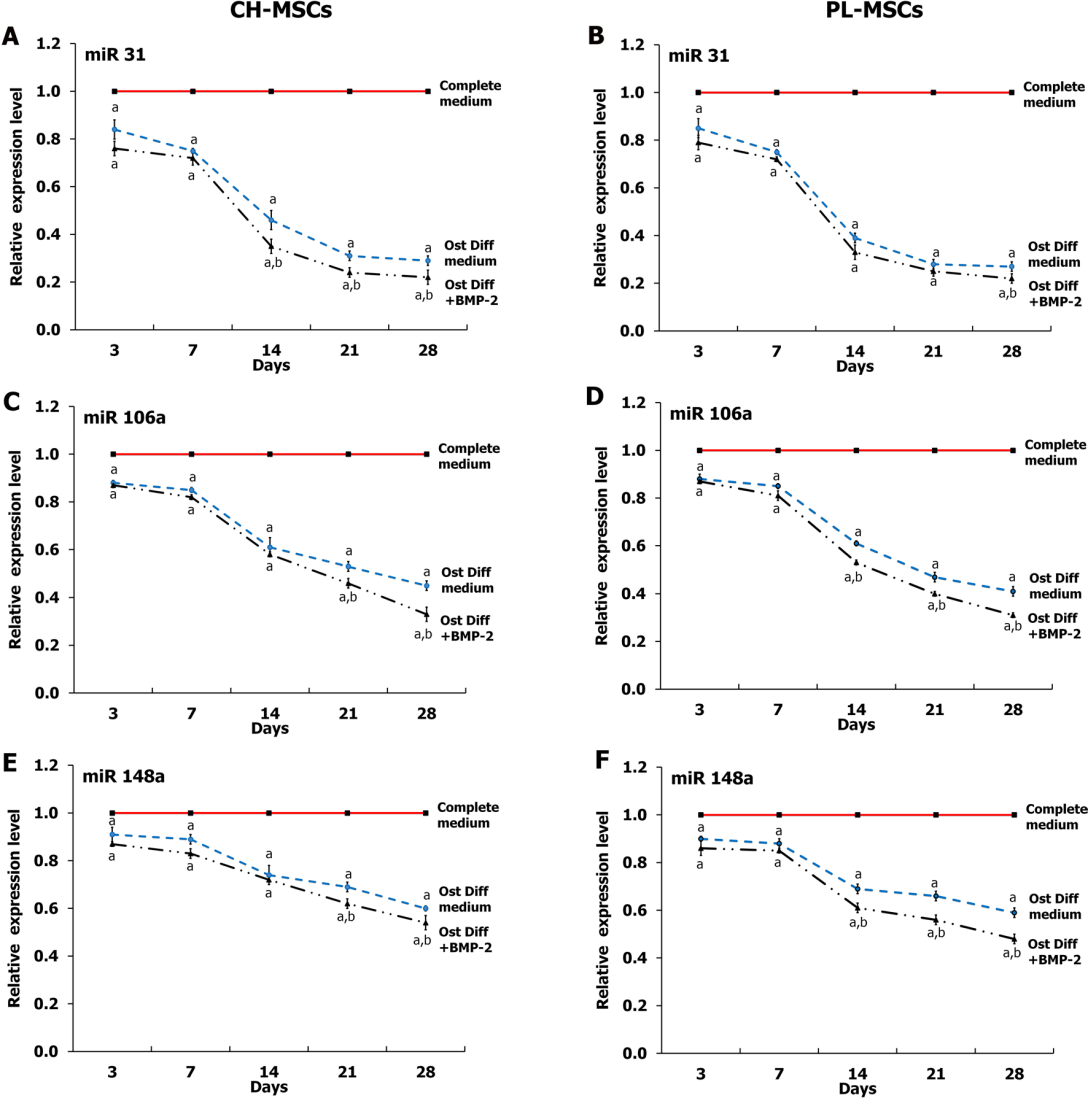
Real-time RT-PCR illustrating the expression of hsa-miR-31, hsa-miR-106a, and hsa-miR-148a during osteogenesis of CH-MSCs (**A**,**C**,**E**) and PL-MSCs (**B**,**D**,**F**) at the indicated time points. Data were calculated based on averages of 3 samples per each condition. *p*<0.05 compared to MSCs cultured in complete DMEM medium marked as “a”. *p*<0.05 compared to MSCs cultured in osteogenic differentiation medium (OstDiff medium) marked as “b”.

**Figure 5:**
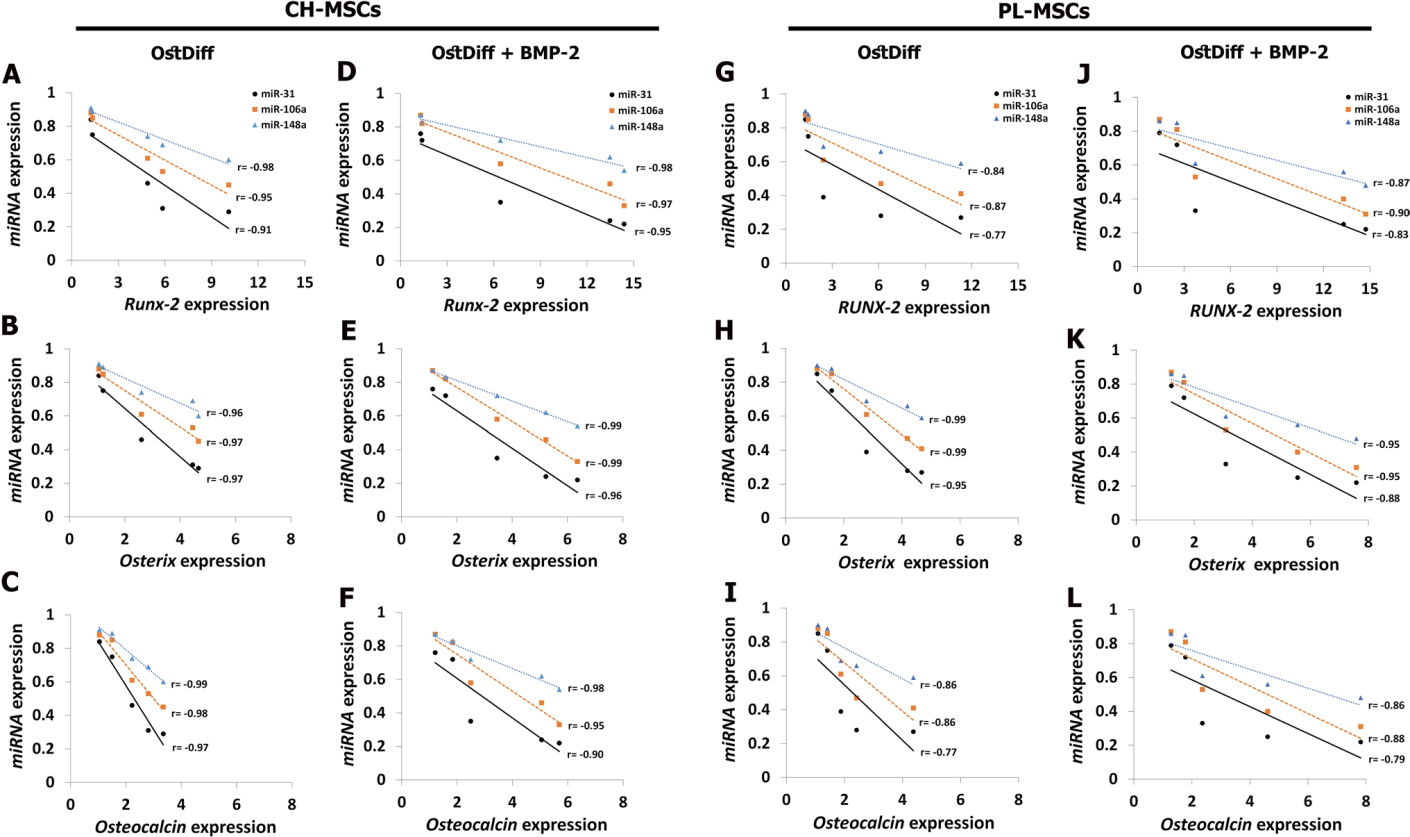
Correlations between the expression of osteogenic marker genes and the expression of miRNAs in CH-MSCs (**A**–**F**) and PL-MSCs (**G**–**L**) cultured in osteogenic differentiation medium with or without BMP-2.

To explore the changes of miRNAs expression during osteogenic differentiation of CH-MSCs and PL-MSCs, 3 miRNAs that are targeted to Runx-2 (hsa-miR-31-5p, hsa-miR-106a-5p, and hsa-miR-148a-5p) were selected. The expression of the miRNAs during osteogenesis was examined using total RNA extracted from CH-MSCs and PL-MSCs that were induced into osteoblasts and compared with untreated control MSCs. The results demonstrated that the expressions of miR-31, miR-106a, and miR-148a were significantly decreased in both CH-MSCs and PL-MSCs cultured in osteogenic induction medium compared with the untreated controls (*P* < 0.05; Fig. 4). Interestingly, the expression of these miRNAs was reduced in a time-dependent manner during the process of osteogenic differentiation of both CH-MSCs and PL-MSCs. The expression of miR-31 significantly decreased in BMP-2 treated CH-MSCs at day 14, 21, and 28 compared to untreated CH-MSCs (*P* < 0.05; Fig. 4A), whereas the expression of miR-31 in BMP-2 treated PL-MSCs show a significant decrease at day 28 compared to untreated PL-MSCs (*P* < 0.05; Fig. 4B). Nevertheless, the expressions of miR-106a and miR-148a in BMP-2 treated CH-MSCs and PL-MSCs showed significant decreases compared to untreated CH-MSCs and PL-MSCs (*P* < 0.05; Fig. 4C–F). It should be noted that the expression of miR-31, miR-106a, and miR-148a were negatively correlated with the expression of osteogenic marker genes in both CH-MSCs and PL-MSCs (Fig. 5).

### The expression levels of miRNAs in CH-MSCs and PL-MSCs in comparison to that of BM-MSCs

**Figure 6:**
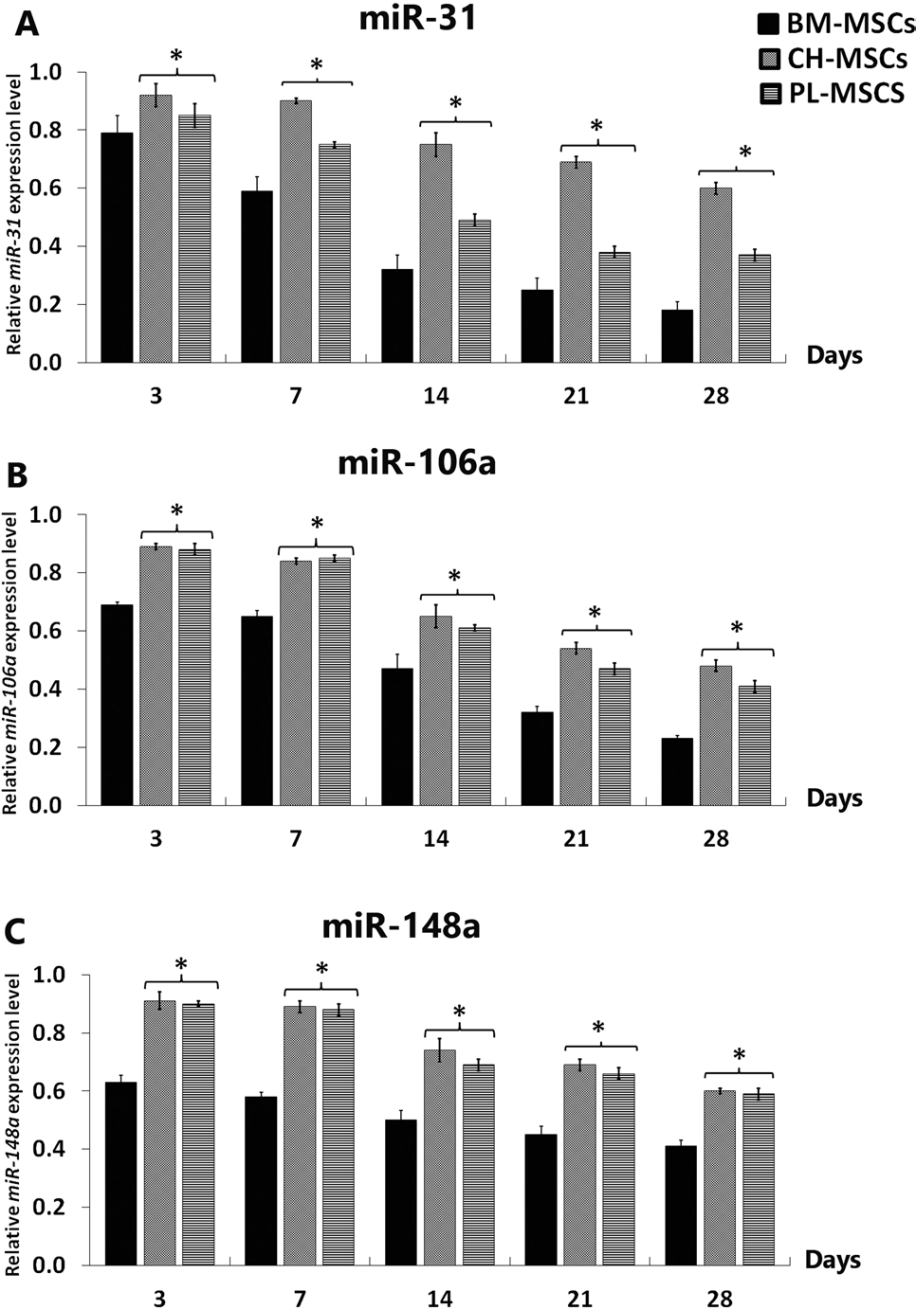
The expression of miR-31, miR-106a, and miR-148a of CH-MSCs and PL-MSCs compared to BM-MSCs. Data were calculated based on averages of 3 samples per each condition. *p<0.05 compared to BM-MSCs.

To correlate the expression levels of specific miRNAs with the osteogenic differentiation capacity of MSCs, the expression levels of miR-31, miR-106a and miR-148a during the osteogenic differentiation of CH-MSCs and PL-MSCs were compared with those of BM-MSCs cultured under the same osteogenic-inducing conditions. Similar to BM-MSCs, the expressions levels of miR-31, miR-106a, and miR-148a were significantly down-regulated during the osteogenic differentiation of CH-MSCs and PL-MSCs in a time-dependent manner. However, the down-regulation of these miRNA expression levels during the osteogenic differentiation of CH-MSCs and PL-MSCs was lesser in extent compared with those of BM-MSCs, resulting in significantly higher levels of miR-31, miR-106a, and miR-148a in CH-MSCs and PL-MSCs in comparison to those of BM-MSCs throughout the osteogenic differentiation process (Fig. 6). These results suggest that the higher expression levels of these miRNAs might be responsible for the lower osteogenic differentiation potential of CH-MSCs and PL-MSCs compared with their bone marrow counterpart.

### Expression of microRNAs after transfection with miRNA inhibitors

**Figure 7:**
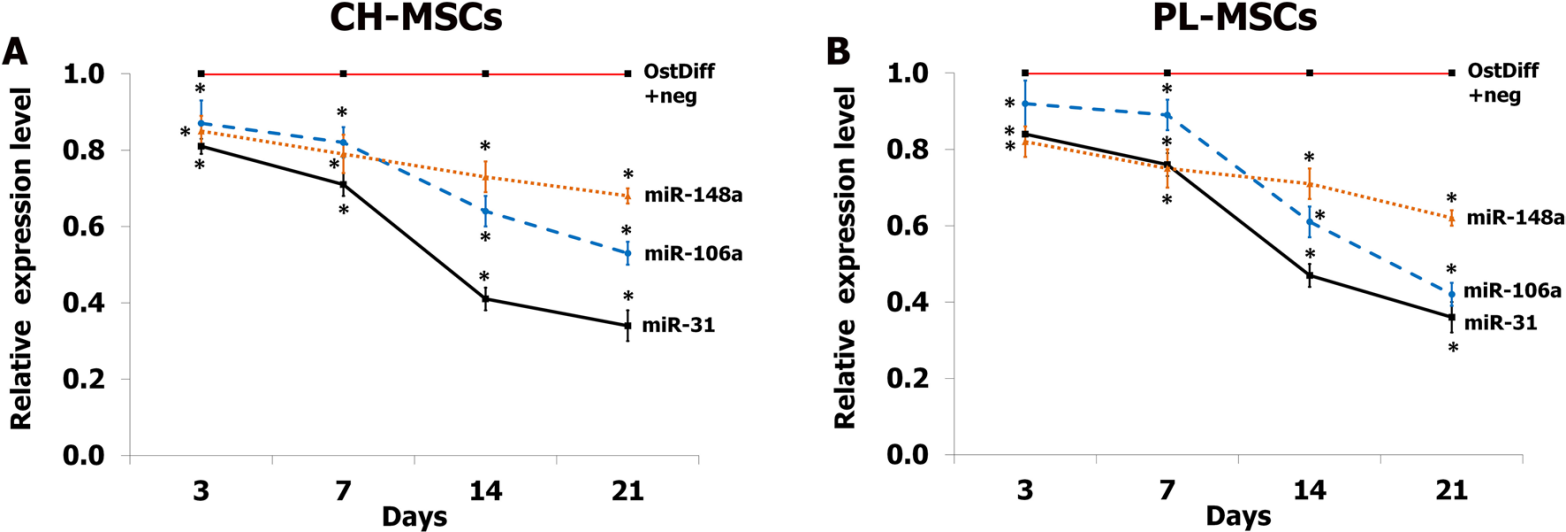
The expression of miR-31, miR-106a, miR-148a in CH-MSCs (**A**) and PL-MSCs (**B**) treated with anti-miR-31, anti-miR-106a, and anti-miR-148a at days 3, 7, 14, and 21. MSCs cultured in osteogenic differentiation medium supplemented with miRNA negative control (OstDiff+Neg) were used as a control. OstDiff+Neg of each time point was used to normalize the data at each individual time point.

To investigate the effect of miRNAs on the osteogenic differentiation potential of CH-MSCs and PL-MSCs, the expression of the miRNAs during osteogenic differentiation was inhibited by transfection with specific miRNA inhibitors. The expression levels of each miRNA after treatment with miRNA inhibitors was examined by quantitative real-time RT-PCR at cultured days 3, 7, 14, and 21. The result revealed that the expression of miR-31 in CH-MSCs and PL-MSCs transfected with anti-miR-31 compared to those transfected with the miRNA negative control was reduced in a time-dependent manner during the process of osteogenic differentiation (Fig. 7). Furthermore, the expressions of miR-106a and miR-148a during osteogenic differentiation of these MSCs were also significantly reduced in a time-dependent manner similar to the expression of miR-31. Although the expression levels of miR-148a in CH-MSCs and PL-MSCs decreased over time, the miR-148a expression levels were not as strongly reduced as those of miR-31 or miR-106a (Fig. 7).

### Expression of alkaline phosphatase after transfection with miRNA inhibitors

**Figure 8:**
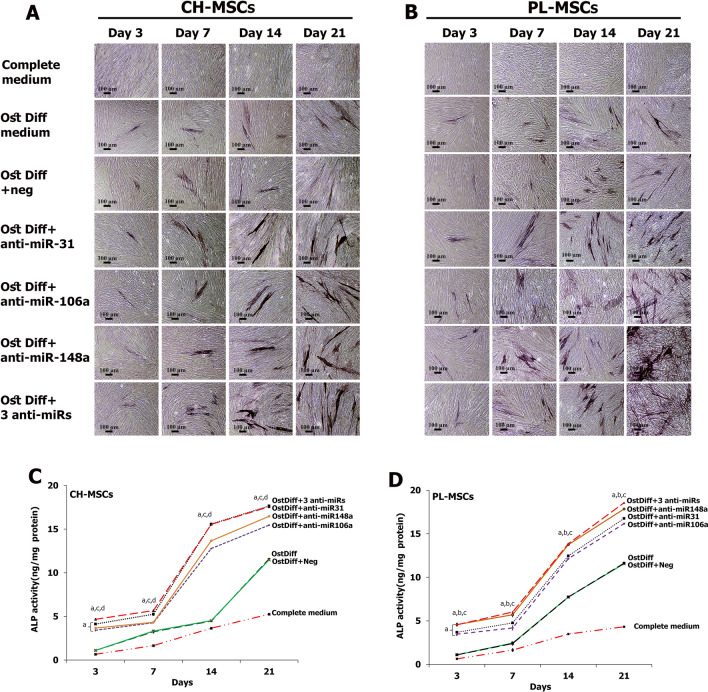
Expression of alkaline phosphatase in CH-MSCs (**A**) and PL-MSCs (**B**) after treatment with anti-miR-31, anti-miR-106a, and anti-miR-148a. The alkaline phosphatase enzyme activity assay showed higher ALP activity in MSCs treated with miRNA inhibitors (**C**,**D**) Statistically significant differences with *p*<0.05 compared to MSCs cultured in osteogenic differentiation medium (OstDiff medium) supplemented with miRNA negative control are marked as “a”. *p*<0.05 compared to MSCs cultured in OstDiff medium supplemented with anti-miR-31, anti-miR-106a or anti-miR-148a are marked as “b”, “c” and “d” respectively. Scale bar = 100 μm.

To analyze the effect of miR-31, miR-106a, and miR-148a on osteogenic differentiation of CH-MSCs and PL-MSCs, anti-miR31, anti-miR106a, and anti-miR148a were applied to down-regulate the action of miR-31, miR-106a, and miR-148a during osteogenic differentiation of CH-MSCs and PL-MSCs. The expression of ALP was examined on days 3, 7, 14, and 21 after transfection. The results revealed that inhibition of each miRNA, anti-miR-31, anti-miR-106a or anti-miR-148a, and inhibition using a combination of all three anti-miRNAs, enhanced the expression of ALP in both CH-MSCs and PL-MSCs compared to MSCs cultured in osteogenic differentiation medium without miRNA inhibitor and MSCs cultured in osteogenic differentiation medium with negative control (Fig. 8). Nevertheless, the combination of the 3 anti-miRNAs did not reveal strong additive or synergistic effects of these anti-miRNAs in ALP expression in either CH-MSCs or PL-MSCs (Fig. 8).

The quantitative assessment of intracellular ALP activity in CH-MSCs and PL-MSCs treated with anti-miRNAs was also performed using colorimetric enzymatic assay on days 3, 7, 14, and 21 after transfection. The results demonstrated that the ALP activity in the anti-miRNA treated groups significantly increased with time. (Fig. 8C, D). As early as 3 days after inhibition, CH-MSCs and PL-MSCs had about a one-fold increase in ALP activity compared to MSCs cultured in the control conditions (complete DMEM, osteogenic differentiation medium both with and without miRNA negative control). On day 14 and day 21, ALP activity in anti-miRNA treated groups was significantly increased up to twofold, compared to the three control MSC groups (*p* < 0.05). Remarkably, PL-MSCs treated with anti-miR-148a or the combination of 3 anti-miRNAs revealed higher ALP activity than PL-MSCs treated with other miRNA inhibitors (*p* < 0.05). However, CH-MSCs treated with anti-miR-31 or the combination of 3 anti-miRNAs revealed higher ALP activity than CH-MSCs treated with other miRNA inhibitors (*p* < 0.05) (Fig. 8C, D).

### Expression of osteogenic genes after transfection with miRNA inhibitors

**Figure 9:**
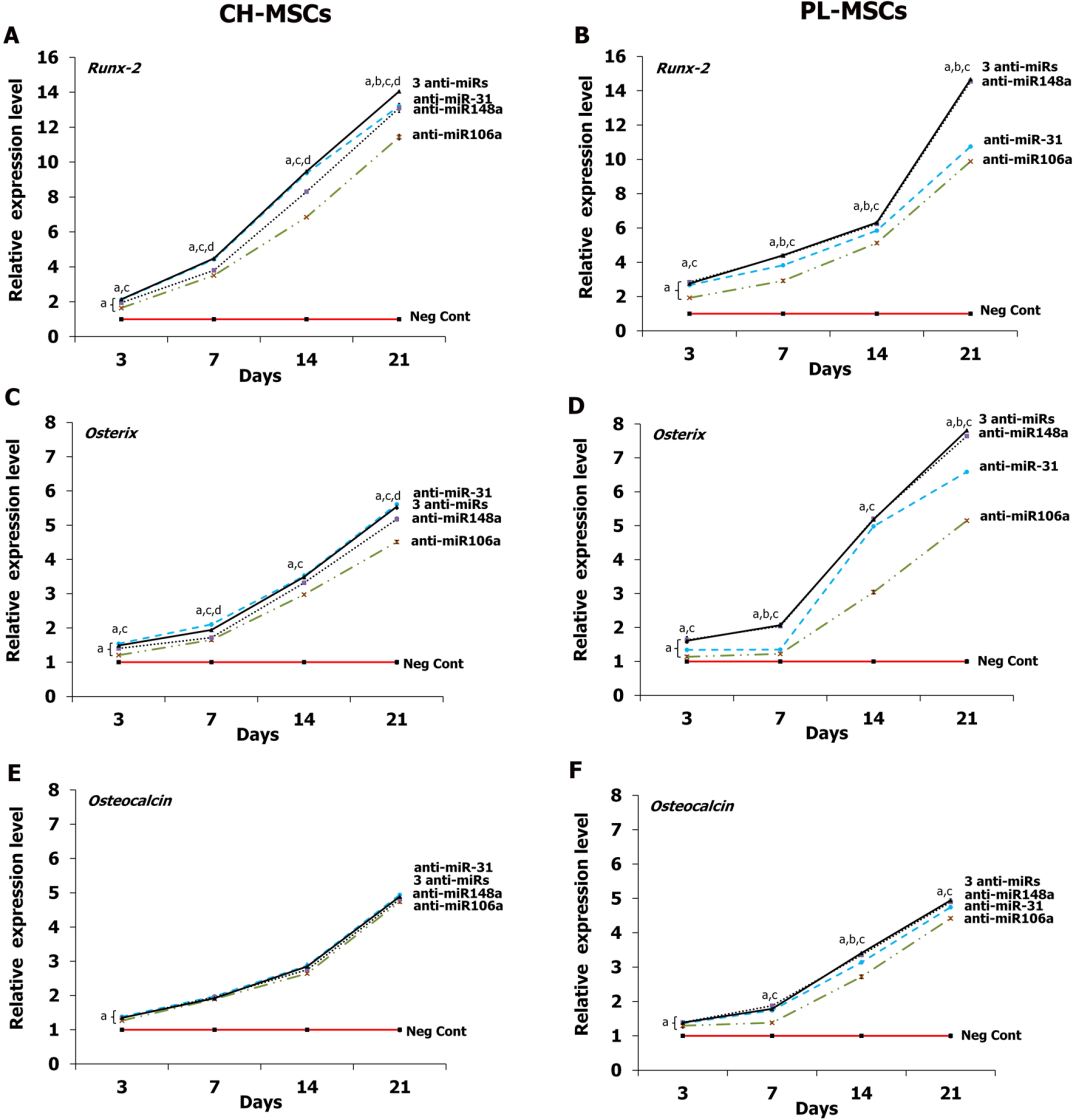
Real-time RT-PCR shows the expression of osteogenic markers in CH-MSCs (**A**,**C**,**E**) and PL-MSCs (**B**,**D**,**F**) after treatment with anti-miR-31, anti-miR-106a, and anti-miR-148a. Statistically significant data with *p*<0.05 compared to MSCs cultured in osteogenic differentiation medium (OstDiff medium) supplemented with miRNA negative control are marked as “a”. *p*<0.05 compared with MSCs cultured in OstDiff medium supplemented with anti-miR-31, anti-miR-106a, or anti-miR-148a are marked as “b”, “c” and “d” respectively.

The effects of miRNA inhibitors on the osteogenic differentiation potential of CH-MSCs and PL-MSCs was further investigated by osteogenic gene expression at day 3, 7, 14, and 21 after anti-miRNA transfections. The results demonstrated that the expressions of Runx-2, Osx, and Ocn increased in a time-dependent manner during the process of osteogenic differentiation of CH-MSCs and PL-MSCs when compared to those transfected with the miRNA negative control (Fig. 9A–F). The expression of Runx-2 in CH-MSCs increased over time from day 3 to day 21. The strongest Runx-2 expression was found in CH-MSCs cultured in osteogenic differentiation medium with anti-miR-31 added or with 3 anti-miRNAs added (Fig. 9A). Nevertheless, CH-MSCs cultured in osteogenic differentiation medium with added anti-miR-106a and anti-miR-148a showed significantly higher Runx-2 expression than those cultured in osteogenic differentiation medium supplemented with miRNA negative control (Fig. 9A). Similar to CH-MSCs, PL-MSCs treated with these anti-miRNAs showed higher Runx-2 expression than the miRNA negative control group. Interestingly, PL-MSCs cultured in osteogenic differentiation medium with added anti-miR-148a or with 3 added anti-miRNAs showed the highest Runx-2 expression (Fig. 9B). The transfections with anti-miR-31, anti-miR-106a, anti-miR-148a, and the combination of 3 anti-miRNAs significantly up-regulated the expression of Osx in CH-MSCs from day 3 onwards (Fig. 9C). Similar to Osx, the lowest Ocn mRNA expression was detected in CH-MSCs on day 3. The Ocn expression levels were significantly increased in CH-MSCs transfected with anti-miR-31, anti-miR-106a, anti-miR-148a, and the combination of 3 anti-miRNAs at day 3, 7, 14, and 21, compared with the miRNA negative control (Fig. 9E, F). The expressions of Ocn were increased to the same extent with time in PL-MSCs treated with anti-miR-31, anti-miR-106a, anti-miR-148a, and the combination of 3 anti-miRNAs (Fig. 9F).

## Discussion

MSCs are a population of multipotent cells, typically isolated from the stromal fraction of bone marrow. Normally, MSCs provide a cellular microenvironment supporting hematopoiesis^27^. They can differentiate into several mesenchymal cell types including osteoblasts, chondrocytes, and adipocytes^28^. In fresh bone marrow, MSCs account for only 0.0001-0.01% of mononuclear cells. Besides, the quantity, proliferation and differentiation potential of MSCs from bone marrow decreases with increasing age^9,29^. Recently, cells exhibiting MSC characteristics have been recognized in various tissues such as adipose tissue, amnion, chorion, placenta, and umbilical cord^10,30^. This study collected chorion and placenta as the alternative sources of MSCs because they can be accessed by a non-invasive procedure. The cells isolated from these tissues exhibited MSC characteristics similar to the cells isolated from bone marrow. During culture, the cells with MSC characteristics selectively adhered to plastic cell culture flasks and exhibited fibroblast-like morphology, whereas the suspended hematopoietic cells were removed through the medium changes. The remaining heterogeneity in cells was progressively eliminated by serial passaging and the self-renewal fraction. Therefore, MSCs were enriched after several passages. The cells isolated from chorion and placenta at passages 3-5, which were further characterized using flow cytometry, were positive for typical MSC markers including CD73, CD90, and CD105 and negative for hematopoietic markers including CD34, CD45 similar to the cells isolataed from bone marrow. These observations are compatible with MSC characteristics^31–33^. Besides, CH-MSCs and PL-MSCs could differentiate into adipocytes and osteoblasts after culture in specific induction media similar to BM-MSCs. The ability of clonally expanded cells to form these distinct cell types is the reliable functional criterion to identify genuine MSCs and distinguish them from pre-osteoblasts and pre-adipocytes which each only give rise to one cell type^34^. Osteogenic differentiation of MSCs is induced by treatment with a pro-osteogenic cocktail consisting of dexamethasone, ascorbic acid-2-phosphate and β-glycerophosphate^35^. Generally, mineralized aggregates can appear after a week in bone marrow-derived MSCs (BM-MSCs), but the treatment is often maintained for up to 3 weeks to maximize the number and size of mineralizing nodules^36^. Different from BM-MSCs, CH-MSCs and PL-MSCs cultured in osteogenic induction medium need more than 4 weeks for differentiation into osteoblasts^37^. Because CH-MSCs and PL-MSCs offer hope for tissue regeneration, an approach for improving osteogenic differentiation potential is a crucial issue^38,39^. Bone morphogenetic protein 2 (BMP-2) is an essential growth factor for bone formation both in vitro and in vivo^40^. It induces the expression of MSC differentiation elements such as DLX5 and Runx-2^41,42^. After treating with BMP-2, both CH-MSCs and PL-MSCs had higher expression of ALP and osteogenic marker genes. This indicates that BMP‐2 treatment overcame the low osteogenic differentiation capacity of both CH-MSCs and PL-MSCs. This is in accordance with a previous study which demonstrated that BMP-2 increased the expression of ALP and osteogenic marker genes in MSCs derived from bone marrow and umbilical cord^43^. A previous study reported that BMP-2 is critically involved in mediating the condensation of mesenchymal cells and direct differentiation to an osteoblastic phenotype^39,44,45^. Overexpression of BMP‐2 promotes the upregulation of osteogenic gene expression, such as ALP and Runx-2 in BM-MSCs^46^. In the process of osteogenic induction, Runx2 plays an important role in the commitment step of osteogenic differentiation^47^. It translocates into the nucleus and acts with Smads through direct binding in a transcriptional activator complex. Runx2 recruits R-Smads to the complex to initiate BMP-responsive gene expression^48^. Above all, BMP‐2 could be a key factor to increase the osteogenic differentiation of CH-MSCs and PL-MSCs.

The elucidation of the underlying molecular mechanisms regulating the osteogenic differentiation of MSCs is important to improve the treatment of bone diseases. Increasing evidence in recent years has demonstrated that miRNAs are an important regulator in osteogenic differentiation of BM-MSCs^25,49^. Dysregulation of miRNA mediated mechanisms is pathologically associated with osteoporosis and other bone diseases^50^. Runx-2 is a key transcription factor associated with osteogenic differentiation. Targeted disruption of Runx-2 results in the maturational arrest of osteoblasts and a complete lack of mineralized bone^51^. A previous study demonstrated that miR-31, miR-106a, and miR-148a were primary inhibitors of osteoblastic differentiation by directly targeting Runx-2^25^. This study demonstrated that miR-31, miR-106a, and miR-148a were downregulated during osteogenic differentiation of CH-MSCs and PL-MSCs. We observed that BMP-2 treated MSCs had lower miRNA expression than non-treated MSCs. Correlation analysis showed that the expression of these miRNAs was negatively correlated with osteogenic gene expression. To further investigate the role of these miRNAs in regulating osteoblastic differentiation of CH-MSCs and PL-MSCs, miRNA inhibitors were added and osteogenic differentiation ability was analyzed. Inhibition of miR-31, miR-106a, and miR-148a promoted osteogenic differentiation of CH-MSCs and PL-MSCs as evidenced by increased ALP activity and osteogenic gene expression. These findings suggest that miR-31, miR-106a, and miR-148a are important in bone formation by negatively regulating the osteogenic differentiation of these MSCs.

A previous study showed that miR-31 was expressed during osteogenic differentiation and mineralization of human BM-MSCs. It modulated the expression of the bone-specific transcription factors during osteogenic differentiation^52^. Interestingly, the expression of miR-31 progressively declined during the osteogenic differentiation of BM-MSCs^53^. Specific miR-31 inhibition increases the expression of Osx, an osteoblast-specific transcription factor, in osteosarcoma cell lines. This also occurred in CH-MSCs and PL-MSCs suggesting that miR-31 regulates the feedback loop that determines osteogenic differentiation of both CH-MSCs and PL-MSCs. Additionally, this regulation coincided with an increase in ALP activity and expression of the osteogenic transcription factors, Runx-2. In skeletogenesis, Runx-2 and Osx are critical transcription factors that play important roles in the cell-fate decision process through which mesenchymal cells become chondrocytes and osteoblasts, through activation of cell type-specific genes. Runx-2, an early differentiation factor, is expressed in the condensed mesenchymal cells. Runx-2-expressing cells keep apart from osteochondroprogenitors to form precursor osteoblasts. Expression of Osx in Runx-2-expressing precursors induces these cells to differentiate into mature osteoblasts, and finally into osteocytes during bone formation^54^. Therefore, the manipulation of Osx expression using miRNA is an appealing option in bone repair.

It has been reported that BMP-2 is the direct target of miR‐106a, and the upregulation of miR‐106a led to the suppression of BMP-2 expression^55^. This study demonstrates that the transfection of anti-miR106a could enhance the osteogenic differentiation capacity of both CH-MSCs and PL-MSCs as evidenced by increased ALP expression and osteogenic gene expression. Downregulation of miR-106a by a miRNA inhibitor increased the osteogenic differentiation potential of both CH-MSCs and PL-MSCs similar to BMP-2 treatment. This supports the hypothesis that miR-106a has a negative regulatory effect on osteogenic lineage specification.

Supplementing miR-148a activity inhibited cell growth and diminished BM-MSCs capacity to differentiate into osteoblast^56^. In contrast, inhibition of miR-148a enhanced the osteogenic differentiation ability of CH-MSCs and PL-MSCs. A previous study showed that overexpression of miR-148a promoted osteoclastogenesis, whereas inhibition of miR-148a attenuated osteoclastogenesis. Mice lacking miR-148a exhibited a significant increase in bone mass^57^. Among 3 miRNA inhibitors, anti-miR-148a showed the highest osteogenic enhancing effect on PL-MSCs. Consequently, PL-MSCs treated with anti-miR148a exhibited the highest ALP activity and the highest expressions of Runx-2 and Osx. On the other hand, CH-MSCs treated with anti-miR-31 exhibited the highest ALP activity and highest expressions of Runx-2 and Osx. These miRNAs, as post-transcriptional regulators of osteogenic differentiation, may serve as novel therapeutic agents for osteogenesis-related disorders.

## Conclusion

MSCs represent a population of cells with the potential to contribute to future treatments of a wide range of degenerative diseases. This study demonstrates that chorion and placenta are good alternative sources of MSCs. Treatment with BMP-2 enhanced osteogenic differentiation potential of CH-MSCs and PL-MSCs by up-regulating the expression of osteogenic genes including Runx-2, Osx, and Ocn. On the other hand, the expression of miR-31, miR-106a, and miR-148a progressively decreased during osteogenic differentiation. The transient transfection with anti-miR31, anti-miR106a, and anti-miR148a increased ALP activity and the expression of osteogenic genes of both CH-MSCs and PL-MSCs. The data suggest that the inhibition of specific miRNAs might be used to enhance the osteogenic differentiation of CH-MSCs and PL-MSCs to supplement the limited supply of BM-MSCs for bone regeneration. However, further in vivo experiments are required to assess the validity of this approach.

## Data Availability

Data sharing not applicable to this article as no datasets were generated or analysed during the current study.

## Abbreviations

ALP: Alkaline phosphatase
BCIP: 5-Bromo-4-chloro-3-indolyl phosphate
BM-MSCs: Bone marrow-derived mesenchymal stem cells
BMP-2: Bone morphogenetic protein 2
CH-MSCs: Chorion-derived mesenchymal stem cells
DLX5: Distal-less homeobox 5
DMEM: Dulbecco’s modified Eagle’s medium
EDTA: Ethylene diamine tetraacetic acid
FBS: Fetal bovine serum
FITC: Fluorescein isothiocyanate
GAPDH: Glyceraldehyde-3-phosphate dehydrogenase
miRNA: MicroRNAs
MSCs: Mesenchymal stem cells
NBT: Nitro blue tetrazolium
Ocn: Osteocalcin
Osx: Osterix
PBS: Phosphate-buffered saline
PDN: Population doubling number
PDT: Population doubling time
PE: Phycoerythrin
PL-MSCs: Placenta derived mesenchymal stem cells
pNPP: P-nitrophenyl phosphate
qRT-PCR: Quantitative real-time polymerase chain reaction
R-Smads: Receptor-regulated Smads
Runx-2: Runt-related transcription factor 2
TGF-β: Transforming growth factor-beta
